# Influence of Fasting Status and Sample Preparation on Metabolic Biomarker Measurements in Postmenopausal Women

**DOI:** 10.1371/journal.pone.0167832

**Published:** 2016-12-08

**Authors:** Neil Murphy, Roni T. Falk, Diana B. Messinger, Michael Pollak, Xiaonan Xue, Juan Lin, Robin Sgueglia, Howard D. Strickler, Mia M. Gaudet, Marc J. Gunter

**Affiliations:** 1 Department of Epidemiology and Biostatistics, School of Public Health, Imperial College London, London, United Kingdom; 2 International Agency for Research on Cancer, Lyon, France; 3 Division of Cancer Epidemiology and Genetics, Hormonal and Reproductive Epidemiology Branch, National Cancer Institute, Bethesda, Maryland, United States of America; 4 The Rockefeller University Hospital, New York, New York, United States of America; 5 Department of Oncology, McGill University, Montreal, Quebec, Canada; 6 Department of Epidemiology and Population Health, Albert Einstein College of Medicine, Bronx, New York, New York, United States of America; 7 Epidemiology Research Program, American Cancer Society, Atlanta, Georgia, United States of America; Shanghai Jiaotong University School of Medicine Xinhua Hospital, CHINA

## Abstract

**Background:**

Epidemiologic data linking metabolic markers-such as insulin, insulin-like growth factors (IGFs)-and adipose tissue-derived factors with cancer are inconsistent. Between-study differences in blood collection protocols, in particular participant’s fasting status, may influence measurements.

**Methods:**

We investigated the impact of fasting status and blood sample processing time on components of the insulin/IGF axis and in adipokines in a controlled feeding study of 45 healthy postmenopausal-women aged 50–75 years. Fasting blood samples were drawn (T0), after which subjects ate a standardized breakfast; subsequent blood draws were made at 1 hour (T1), 3 hours (T3), and 6 hours (T6) after breakfast. Serum samples were assayed for insulin, C-peptide, total- and free-IGF-I, IGF-binding protein [BP]-1 and -3, total and high molecular weight (HMW)-adiponectin, retinol binding protein-4, plasminogen activator inhibitor (PAI)-1, and resistin.

**Results:**

Insulin and C-peptide levels followed similar postprandial trajectories; intra-class correlation coefficients [ICC] for insulin = 0.75, (95%CI:0.64–0.97) and C-peptide (ICC = 0.66, 95%CI:0.54–0.77) were similarly correlated in fasting (Spearman correlation, *r* = 0.78, 95%CI:0.64–0.88) and postprandial states (T1, *r* = 0.77 (95%CI: 0.62–0.87); T3,*r* = 0.78 (95%CI: 0.63–0.87); T6,*r* = 0.77 (95%CI: 0.61–0.87)). Free-IGF-I and IGFBP-1 levels were also affected by fasting status, whereas total-IGF-I and IGFBP-3 levels remained unchanged. Levels of adipokines were largely insensitive to fasting status and blood sample processing delays.

**Conclusion:**

Several components of the insulin/IGF axis were significantly impacted by fasting state and in particular, C-peptide levels were substantially altered postprandially and in a similar manner to insulin.

## Introduction

Obesity, Type 2 diabetes (T2D) and metabolic syndrome are significant risk factors for cancers at multiple sites including postmenopausal breast, colorectum, endometrium, liver, kidney and pancreas [1–3]. A growing number of epidemiological studies have investigated the biological pathways which may link obesity, T2D, and cancer risk. Insulin/insulin-growth factor-1 (IGF-1) axis markers and abnormal levels of adipokines have been implicated as underlying, in part, the obesity-cancer relationship. However, for common malignancies, such as postmenopausal breast cancer and colorectal cancer, there are inconsistencies in the literature and some prospective investigations have reported conflicting findings [4–9].

For example, in an analysis conducted within the Women’s Health Initiative Observational Study, higher fasting insulin levels were associated with greater breast cancer risk [4], however, other studies which measured C-peptide levels as a marker of insulin secretion, often in non-fasting bloods, have generally reported null relationships [5,6]. As a further example, adiponectin, an insulin-sensitizing adipokine which has anti-inflammatory activities, has been inversely associated with colorectal cancer risk in some [7,8], but not all [9], prospective investigations.

A possible contributory factor to the observed heterogeneous results across studies is between–study differences in blood collection protocols. For instance, it is unclear how the time interval between last meal and blood draw (i.e., fasting status) affects measurement of various metabolic parameters such as the insulin/IGF axis and adipokines. In addition, it is unknown how the separation of serum from whole blood (i.e. processing time) influences measured adipokines levels. The effects of blood specimen processing time on measured levels of IGF axis markers has previously been investigated and showed, for example, that total IGF-I and IGFBP-3 levels were unaffected by variation in sample processing while free IGF-I levels changed significantly [10].

To investigate how circulating levels of insulin/IGF axis components and adipokine levels change in response to the transition from fasting to non-fasting state and sample processing, we conducted a controlled feeding study in postmenopausal women with sequential blood draws and measured levels of insulin, C-peptide, total IGF-I, free IGF-I, IGF-binding protein-1 (IGFBP-1), and IGF-binding protein-3 (IGFBP-3), as well as levels of adipokines, including, total and high molecular weight (HMW) adiponectin, retinol binding protein-4 (RBP-4), plasminogen activator inhibitor-1 (PAI-1), and resistin. Understanding the role of fasting status and blood sample processing time, using a study design that is modeled after a typical epidemiologic study conducted among healthy women, could inform the design, analysis, and interpretation of epidemiologic studies of these obesity-related serological markers.

## Materials and Methods

### Study Subjects

Study subjects were 45 postmenopausal women aged 50–75 years old who were recruited via advertisements directed to the existing Rockefeller University healthy volunteer database, the Rockefeller University campus, local medical centers, and senior centers. Criteria for inclusion in the study were: no use of tobacco products in the previous 6 months; non-users of exogenous hormones in the previous 6 months, including hormone replacement therapy (HRT); and, not taking thyroid medication at the time of blood draw. In addition, women with diabetes, thyroid conditions, and glucose levels above 180 mg/dl at the screening visit were not eligible for inclusion. Study participants were recruited at the Center for Clinical and Translational Science (CCTS) at the Rockefeller University Hospital (New York, NY). Women who completed the study intervention received 200 USD in compensation. The Institutional Review Board at Rockefeller University reviewed and approved all phases of the study.

### Study Intervention

Subjects arrived at the study center at 8:00AM (+/- 30 minutes) and had fasted overnight (for at least 8 hours). Blood was drawn at four time intervals. The first blood draw (T0)–based on an overnight fast—included 3x10ml red-top tubes (with clot activator for serum separation) of blood and a glucose finger stick test. A research breakfast was then given to the subjects and was expected to be consumed entirely within 20 minutes. The total calories of the breakfast were adjusted to 1/3 of the subject’s estimated daily energy needs for weight maintenance (based on the Harris-Benedict equation for resting energy expenditure). The meal mimicked the composition of an average U.S. diet (i.e., 15% protein, 32–33% fat and 52–53% carbohydrate). As an example, a subject requiring 2,100 calories per day for weight maintenance was served a metabolic breakfast meal with 700 calories, and contained ½ cup orange juice, 1½ oz cornflakes, ½ cup whole milk, ½ cup light yogurt, and ½ oz chopped walnuts. Lactose-free milk was substituted for regular milk for one subject who was lactose-intolerant.

After the breakfast was consumed, postprandial blood samples were drawn 1 hour after completion of the meal (T1) and again at hour 3 (T3) and hour 6 (T6). For the T1 and T3 blood draws, 3x10ml red-top tubes of blood (with clot activator for serum separation) were collected, while 6x10ml red-top tubes of blood (with clot activator for serum separation) were collected for T6. Between the research breakfast and the three postprandial blood draws, the study subjects were under observation by study nurses and were able to walk around the study center and use its recreational facilities (e.g., computers and TVs/DVDs). During this period, subjects were prohibited from certain activities (e.g., heavy lifting and running) and from consuming any foods (with the exception of water) until after the last blood draw (T6).

### Blood Processing

Following blood draw, the blood specimens were immediately stored on ice. Within 30 minutes of the T0, T1, and T3 draws, the tubes were centrifuged. Serum was then aliquoted and stored at -80°C. The 6x10ml red-top tubes from the T6 draw were processed in sets of two. The first set was processed immediately (T6P0). The two remaining sets were stored at 4°C for 24 hours (T6P24) and 48 hours (T6P48) before processing.

### Laboratory Assays

Serum samples were assayed for insulin and C-Peptide by RIA (Linco Products, now known as EMD Millipore). Serum levels of free IGF-1 were quantified by RIA (Diagnostic Systems Laboratory), while levels of total IGF-1, IGFBP-1, and IGFBP-3 were quantified by ELISA (ALPCO Diagnostics). Serum samples were assayed in duplicate for adipokines, including total and HMW adiponectin, RBP-4, PAI-1, and resistin, in Dr. Michael Pollak’s laboratory using commercially-available ELISA-based assays for each adipokine (EMD Millipore). In addition to the 270 study samples (45 women x 6 samples/ woman at T0, T1, T3, T6P0, T6P24, and T6P48), 20 blinded quality control (QC) samples were included. The QC samples were plated so that there were 2 sets of replicates in each batch to estimate within and between batch variations. For all of the insulin/IGF axis and adipokines measurements, except resistin, inter- and intra-batch CVs were <10%. For resistin, the intra-batch CV was 1.6% and the inter-batch was 21.4%.

### Statistical Analysis

Changes in serological measurements from the fasting (T0) level for each serological factor were evaluated at each postprandial blood draw (T1, T3, and T6), separately. Several of the serological markers had skewed distributions (total adiponectin, HMW adiponectin, total IGF-I, IGFBP-3, IGFBP-1, and resistin). The medians and median absolute deviations (MAD) for each biomarker were plotted for each time period. Intraclass correlation coefficients (ICC), defined as the ratio of between-subject variance to total variance, were calculated as a measure of the reproducibility of each serological factor over time within subjects. We conducted analyses for all women combined, and by body mass index (BMI) strata (normal weight 18.5-<25 kg/m^2^; overweight 25-<30 kg/m^2^; and obese ≥30 kg/m^2^).

## Results

In this population of postmenopausal women, the median age among subjects was 62 years (interquartile range [IQR]: 57–66 years) (Table 1). The median BMI of the 45 subjects was 26.6 kg/m^2^ (IQR: 23.9–31.1 kg/m^2^). A Spearman’s correlation matrix for the insulin/IGF-1 axis parameters and adipokines measured on blood samples collected 1 hour after the research breakfast is presented in Table 2.

**Table 1:** Characteristics of study subjects.

	All women *(n = 45)*	
**Age (years)** *	62.0	(57.0–66.0)
**Race/ethnicity**		
White/non-Hispanic *(n = 29)*		64.4%
White/Hispanic *(n = 4)*		8.9%
Black *(n = 10)*		22.2%
Other *(n = 2)*		4.5%
**Body mass index (kg/m**^**2**^**)**	26.6	(23.9–31.1)
Normal weight (18.5-<25.0) *(n = 14)*		31.1%
Overweight (25.0-<30.0) *(n = 17)*		37.8%
Obese (≥30.0) *(n = 14)*		31.1%
**Waist circumference (cm)** *	94.5	(83.0–106.5)
**Hip circumference (cm)** *	106.1	(98.3–118.0)
Insulin/IGF-1 axis fasting parameters levels*		
C-peptide (ng/ml)	1.9	(1.5–2.3)
Insulin (uU/ml)	15.0	(11.7–20.8)
Total-IGF-1 (ng/ml)	165.0	(135.1–197.6)
Free-IGF-1 (ng/ml)	0.6	(0.42–0.93)
IGFBP1 (ug/l)	5.8	(2.9–11.7)
IGFBP3 (ug/ml)	32.4	(28.0–35.1)
Adipokines fasting levels*		
Adiponectin (ng/ml)	11803.7	(7328.1–18674.9)
HMW Adiponectin (ng/ml)	6144.2	(2810.5–10000.3)
Resistin (ng/ml)	8.5	(6.7–10.1)
PAI-1 (ng/ml)	8.7	(6.8–10.8)
RBP-4 (ng/ml)	39233.5	(36372.0–45006.5)

* Median and interquartile (IQR) range.

**Table 2:** Spearman’s correlation matrix for the insulin/IGF-1 axis parameters and adipokines measured on blood samples collected 1 hour after the research breakfast (T1).

	**C-peptide**	**Insulin**	**Total-IGF-1**	**Free-IGF-1**	**IGFBP1**	**IGFBP3**	**Adiponectin**	**HMW Adiponectin**	**Resistin**	**PAI-I**	**RBP-4**
**C-peptide**	1										
**Insulin**	0.77	1.00									
**Total-IGF-1**	-0.31	-0.25	1.00								
**Free-IGF-1**	-0.29	-0.0002	0.29	1.00							
**IGFBP1**	-0.27	-0.49	-0.12	-0.35	1.00						
**IGFBP3**	-0.10	-0.09	0.43	0.17	0.15	1.00					
**Adiponectin**	-0.44	-0.52	0.13	0.07	0.63	0.06	1.00				
**HMW Adiponectin**	-0.44	-0.48	0.11	0.12	0.56	0.03	0.98	1.00			
**Resistin**	0.02	0.06	-0.22	-0.09	-0.06	-0.11	-0.22	-0.24	1.00		
**PAI-I**	0.42	0.35	-0.07	-0.09	-0.24	0.10	-0.41	-0.37	0.29	1.00	
**RBP-4**	0.02	0.01	0.07	-0.02	0.08	0.06	-0.004	-0.05	0.19	0.20	1.00

**Fig 1:**
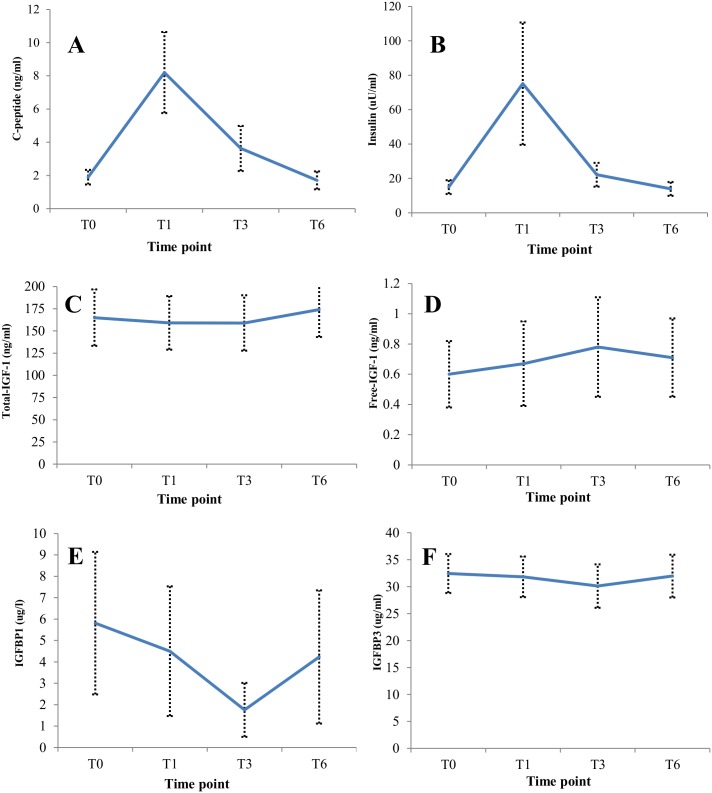
Medians and median absolute deviation of serum (A) C-peptide, (B) insulin, (C) total IGF-1, (D) free-IGF-1, (E) IGFBP-1, and (F) IGFBP-3 after an overnight fast (T0), and 1 hour (T1), 3 hours (T3), and 6 hours (T6) after the research breakfast.

Serum values of C-peptide and insulin followed similar postprandial trajectories, rising steeply between T0 and T1 and returning to approximately fasting levels by T6 (Fig 1). Similar ICC values between the four time points were observed for insulin (ICC = 0.75, 95% CI: 0.64–0.97) and C-peptide (ICC = 0.66, 95% CI: 0.54–0.77) (Table 3). The Spearman correlations of insulin and C-peptide levels at the four time points were: T0 (fasting) = 0.78 (95% CI: 0.64–0.88); T1 = 0.77 (95% CI: 0.62–0.87); T3 = 0.78 (95% CI: 0.63–0.87); and T6 = 0.77 (95% CI: 0.61–0.87). Serum levels of total-IGF-1 (ICC = 0.96, 95% CI: 0.93–0.97), and IGFBP-3 (ICC = 0.94, 95% CI: 0.90–0.96) were stable between fasting and postprandial time points (Fig 1; Table 3). However, free-IGF-1 levels rose in the immediate postprandial phases (T1 and T3), before declining by T6 (ICC = 0.54, 95% CI: 0.40–0.68). IGFBP-1 levels declined during the immediate postprandial phase, before rising by T6 (ICC = 0.85, 95% CI: 0.78–0.90) (Fig 1; Table 3). These insulin/IGF-1 axis results were similar for normal weight, overweight, and obese women (data not shown).

**Table 3:** Medians of insulin/IGF-1 axis parameters and adipokines after an overnight fast (T0), and 1 hour (T1), 3 hours (T3), and 6 hours (T6) after the research breakfast, and intraclass correlations between measurements on blood samples collected at all time-points.

	T0	T1	*% diff* *T0 to T1*	T3	*% diff* *T1 to T3*	T6	*% diff* *T3 to T6*	Intraclass correlation (ICC)^‡^	95% CI
**Insulin/IGF-1 axis parameters**									
C-peptide (ng/ml)	1.9	8.2	*331*.*6%*	3.6	*-55*.*7%*	1.7	*-52*.*9%*	0.66	0.54–0.77
Insulin (uU/ml)	15.0	75.1	*402*.*2%*	22.1	*-70*.*6%*	13.9	*-37*.*3%*	0.75	0.64–0.97
Total-IGF-1 (ng/ml)	165.0	159.1	*-3*.*6%*	159.0	*-0*.*1%*	174.1	*9*.*5%*	0.96	0.93–0.97
Free-IGF-1 (ng/ml)	0.6	0.7	*11*.*7%*	0.8	*16*.*4%*	0.7	*-9*.*0%*	0.54	0.40–0.68
IGFBP1 (ug/l)	5.8	4.5	*-22*.*5%*	1.8	*-60*.*9%*	4.2	*140*.*3%*	0.85	0.78–0.90
IGFBP3 (ug/ml)	32.4	31.8	*-1*.*9%*	30.1	*-5*.*4%*	32.0	*6*.*1%*	0.94	0.90–0.96
**Adipokines fasting levels**									
Adiponectin (ng/ml)	11803.7	12425.3	*5*.*3%*	12418.3	*-0*.*1%*	12015.1	*-3*.*2%*	0.99	0.98–0.99
HMW Adiponectin (ng/ml)	6144.3	6215.1	*1*.*2%*	5931.7	*-4*.*6%*	6214.8	*4*.*8%*	0.99	0.98–0.99
Resistin (ng/ml)	8.5	9.0	*6*.*2%*	9.3	*3*.*7%*	8.8	*-5*.*5%*	0.85	0.77–0.90
PAI-I (ng/ml)	8.7	8.1	*-6*.*0%*	7.6	*-6*.*6%*	6.8	*-10*.*5%*	0.88	0.82–0.92
RBP-4 (ng/ml)	39233.5	39157.5	*-0*.*2%*	38253.0	*-2*.*3%*	39923.5	*4*.*4%*	0.93	0.89–0.95

^**‡**^ Between measurements on blood samples collected at fasting (T0), and 1 hour (T1), 3 hours (T3), and 6 hours (T6) after the research breakfast.

**Fig 2:**
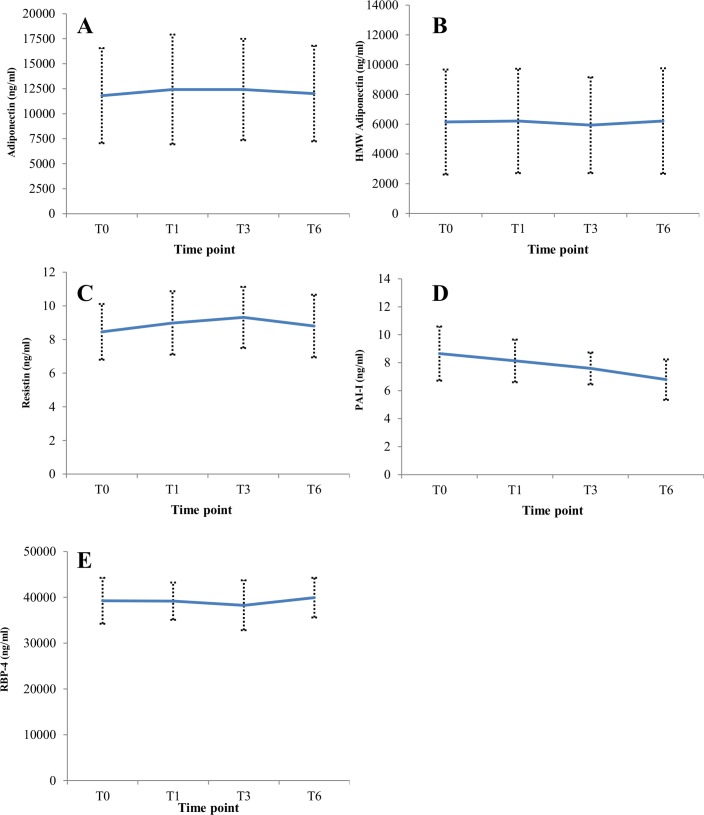
Medians and median absolute deviation (MAD) of serum (A) adiponectin, (B) HMW adiponectin, (C) resistin, (D) PAI-I, and (E) RBP-4 after an overnight fast (T0), and 1 hour (T1), 3 hours (T3), and 6 hours (T6) after the research breakfast.

Adiponectin (ICC = 0.99, 95% CI: 0.98–0.99) and HMW adiponectin (ICC = 0.99, 95% CI: 0.98–0.99) were stable over the four time points (T0, T1, T3, and T6) (Fig 2; Table 3). The 24 hour and 48 hour delay in processing the blood samples did not affect the stability of adiponectin (ICC = 0.99, 95% CI: 0.98–0.99) and HMW adiponectin (ICC = 0.99, 95% CI: 0.98,0.99).

Levels of resistin (ICC = 0.81, 95% CI: 0.71–0.87) and RBP-4 (ICC = 0.92, 95% CI: 0.88–0.95) were stable over the four time-points, and were insensitive to the 24 hours and 48 delays in blood sample processing (data not shown). Levels of PAI-1 decreased with greater time since last meal (Fig 2; Table 3); however, the ICC across the four T0-T6 time points was 0.88 (95% CI: 0.82–0.92), and levels were insensitive to the 24 hours and 48 delays in blood sample processing (data not shown). These adipokines results were similar for normal weight, overweight, and obese women (data not shown).

## Discussion

In a controlled feeding study of 45 healthy postmenopausal women, we explored the impact of change in fasting status and sample processing time on serum levels of insulin/IGF axis components and adipokines. Our results suggest that insulin and C-peptide levels are similarly correlated in fasting and postprandial states and follow very similar trajectories following a meal. Free IGF-I and IGFBP-1 levels were also affected by fasting status while total IGF-I and IGFBP-3 levels remained stable. Adipokines levels were largely insensitive to fasting status and delays in blood sample processing. Our results suggest that in epidemiological studies which collect blood specimens in the non-fasting state, failure to control for time since last meal (e.g. adopting a case-control study design and matching by time since last meal) could have substantial impact on observed associations between insulin, C-peptide, free IGF-I, and IGFBP-1 with disease outcomes.

The measurement of C-peptide as a marker of insulin secretion, rather than insulin itself, has been preferred in many epidemiological studies [5,11,12]. This is mainly because C-peptide and insulin are co-secreted in equimolar concentrations, and C-peptide has a slower metabolic clearance rate than insulin and as such, may be less sensitive to changes in fasting status [13]. However, in our analysis, C-peptide and insulin followed near-identical postprandial increases in concentrations before reducing to fasting levels 6 hours after food intake. This suggests that C-peptide is not necessarily a more stable marker of insulin levels in the non-fasting state. Indeed, similar Spearman correlations (r = 0.77 to 0.78) were found between C-peptide and insulin levels for the fasting (T0) and postprandial (T1, T3, and T6) measurements. Overall, our data highlight the importance of fasting blood samples being used to measure both insulin markers and that C-peptide should not necessarily be considered as a superior or more stable indicator of insulin secretion for future epidemiological investigations in healthy postmenopausal women.

Insulin is a major regulator of IGFBP-1, acting to reduce its expression [14,15], hence the decline in IGFBP-1 levels we observed in the immediate postprandial phases were likely a consequence of this regulatory mechanism. Such postprandial declines in IGFBP-1 levels would also lead to the increased availability of free-IGF-1, explaining the immediate postprandial increases in free-IGF-1 levels observed in this study.

Previous controlled feeding studies for adipokines have usually focused on the postprandial effects of extreme diets or in subjects with endocrine disorders [16,17]. Our study design was modelled after a typical epidemiological investigation conducted among healthy postmenopausal women, and our results can therefore be informative for the design and interpretation of cohort studies where blood specimens are collected prior to disease diagnosis. Our results demonstrated that adiponectin, HMW adiponectin, resistin, and RBP-4 are very stable biomarkers that seem unaffected by fasting status, findings generally consistent with previous controlled feeding studies [17–20]. For PAI-I, and in line with previous studies, levels progressively decreased postprandially [21,22], which suggests that epidemiological study participants should ideally have fasted for at least 6 hours when measuring PAI-I. To our knowledge, this was the first investigation on the impact of blood sample processing on adipokines levels, and our results indicate that these metabolic biomarkers can be reliably measured regardless of sample processing delay allowing flexibility to blood sample processing protocols of future studies measuring adipokines.

Limitations of our study include possible lack of generalizability of the results to populations beyond healthy postmenopausal women. In addition, it is possible that certain dietary meal compositions may stimulate different post-prandial effects. Further, it is possible other unmeasured factors contributed to variability in the serologic factors assayed in this analysis. For example circadian or biorhythms may vary between individuals and could have impacted levels of the metabolic markers as well as their postprandial trends [23]. Finally, use of the Harris-Benedict equation to determine basal metabolic rate may have resulted in the under-estimation of the calorie requirements for overweight and obese women; however, we do not believe this would have influenced our findings as our results were consistent across all body sizes.

In summary, the results of this controlled feeding study suggest that the measurements of C-peptide and insulin are equivalent in both fasting and postprandial states, and that adipokines levels are generally insensitive to fasting status and delays in blood sample processing. Future epidemiological studies which measure insulin markers should be conducted in fasting study subjects only or be finely controlled for time since last meal (e.g. use of a matched case-control study design by fasting time).

## Supporting Information

S1 File Raw subject level data of all studied variables (data in csv format). (CSV)
